# Evaluating the Artificial Intelligence Performance Growth in Ophthalmic Knowledge

**DOI:** 10.7759/cureus.45700

**Published:** 2023-09-21

**Authors:** Cheng Jiao, Neel R Edupuganti, Parth A Patel, Tommy Bui, Veeral Sheth

**Affiliations:** 1 Ophthalmology, Augusta University Medical College of Georgia, Augusta, USA; 2 Neurology, Augusta University Medical College of Georgia, Augusta, USA; 3 Ophthalmology, University Retina and Macula Associates, Oak Forest, USA

**Keywords:** chatgpt, ophthalmology, medical education, natural language processing models, artificial intelligence

## Abstract

Objective: We aim to compare the capabilities of Chat Generative Pre-Trained Transformer (ChatGPT)-3.5 and ChatGPT-4.0 (OpenAI, San Francisco, CA, USA) in addressing multiple-choice ophthalmic case challenges.

Methods and analysis: Both models’ accuracy was compared across different ophthalmology subspecialties using multiple-choice ophthalmic clinical cases provided by the American Academy of Ophthalmology (AAO) “Diagnosis This” questions. Additional analysis was based on image content, question difficulty, character length of models’ responses, and model’s alignment with responses from human respondents. χ2 test, Fisher’s exact test, Student’s t-test, and one-way analysis of variance (ANOVA) were conducted where appropriate, with p<0.05 considered significant.

Results: GPT-4.0 significantly outperformed GPT-3.5 (75% versus 46%, p<0.01), with the most noticeable improvement in neuro-ophthalmology (100% versus 38%, p=0.03). While both models struggled with uveitis and refractive questions, GPT-4.0 excelled in other areas, such as pediatric questions (82%). In image-related questions, GPT-4.0 also displayed superior accuracy that trended toward significance (73% versus 46%, p=0.07). GPT-4.0 performed better with easier questions (93.8% (least difficult) versus 76.2% (middle) versus 53.3% (most), p=0.03) and generated more concise answers than GPT-3.5 (651.7±342.9 versus 1,112.9±328.8 characters, p<0.01). Moreover, GPT-4.0’s answers were more in line with those of AAO respondents (57.3% versus 41.4%, p<0.01), showing a strong correlation between its accuracy and the proportion of AAO respondents who selected GPT-4.0’s answer (ρ=0.713, p<0.01).

Conclusion and relevance: Our study demonstrated that GPT-4.0 significantly outperforms GPT-3.5 in addressing ophthalmic case challenges, especially in neuro-ophthalmology, with improved accuracy even in image-related questions. These findings underscore the potential of advancing artificial intelligence (AI) models in enhancing ophthalmic diagnostics and medical education.

## Introduction

The intersection of artificial intelligence (AI) and healthcare presents opportunities for improved clinical decision-making and medical education. The rising competency of AI in diagnosing diverse ocular conditions underscores its potential within ophthalmology [1,2]. In this field, natural language processing (NLP) models, notably the Generative Pre-trained Transformer (GPT) series developed by OpenAI (San Francisco, CA, USA), have brought promising potential for language understanding and healthcare diagnostics [3,4]. GPT models are trained on a textual database, and they learn to generate coherent and contextually appropriate responses based on the abstract relationship between words (tokens) within the neural network [5]. Previous studies show that GPT-3.5 achieves over 50% accuracy on the United States Medical Licensing Examination (USMLE), nearing the 60% pass mark. Furthermore, over 90% of AI responses provided substantial insights, demonstrating deductive reasoning that could be beneficial for human learners [6]. The GPT models’ capabilities extend beyond general medical education. For example, when tested with practice ophthalmic board questions, ChatGPT was able to answer 46% of them correctly in January 2023 [7].

Since then, the GPT model has been updated from GPT-3.5 to GPT-4.0, showcasing potential enhancements in AI-enabled medical education. The new model developed better contextual understanding, enhanced topic consistency, and markedly increased accuracy as evidenced by its success on professional and academic assessments, improving from the 10th percentile to the 90th percentile on the Uniform Bar Exam [8]. Recent studies utilizing the Basic and Clinical Science Course (BCSC) Self-Assessment Program for the Ophthalmic Knowledge Assessment Program (OKAP) examination have shown vast improvements in recent ChatGPT iterations that can match the accuracy rate of human respondents [9]. Yet, the AI performance growth seen in the GPT models, particularly in non-board style ophthalmology clinical cases, remains empirically under-examined. Our study compares the capabilities of ChatGPT-3.5 and ChatGPT-4.0 using ophthalmic case challenges.

## Materials and methods

Clinical ophthalmology questions were sourced from the 2022 release of the American Academy of Ophthalmology (AAO) “Diagnose This,” a compendium of weekly clinical ophthalmology multiple-choice questions [10]. We chose AAO “Diagnose This” due to its focus on clinical decision-making rather than conventional standardized testing. The 2022 release ensured the exclusion of any data indexed by GPT models trained before January 1, 2022. Because the study exclusively utilized public data and did not involve human participants, it adhered to the American Association for Public Opinion Research (AAPOR) reporting guidelines and did not require ethical clearance.

The primary objective of the study was to compare the accuracy of GPT-3.5 and GPT-4.0 across different subspecialties of ophthalmology. The secondary objectives included evaluating performance on image-related questions, varying question difficulty, character length of model responses, and alignment with responses from AAO respondents.

All 52 questions from the 2022 AAO “Diagnose This” were categorized based on the specified ophthalmology subspecialty: cornea/anterior segment, glaucoma, neuro-ophthalmology, ocular pathology/oncology, oculoplastic/orbit, pediatric, refractive, retina, and uveitis. Opening and utilizing a premium OpenAI account with no prior usage, we ensured zero preceding conversation history. Questions and corresponding choices were inputted directly into GPT-3.5 and GPT-4.0 without initial prompts. To maintain the integrity of responses, we cleared each prior ChatGPT conversation since the model’s replies can be shaped by ongoing dialogues. Example input and outputs can be referenced in the Results. Responses were assessed for accuracy by checking the selected answer from GPT outputs with AAO’s correct answer. Any ambiguity in model choices triggered a re-prompt, instructing them to “choose one of the following options.”

For the secondary analysis, we aimed to analyze ChatGPT’s performance based on not only the subject but also the level of difficulty, type of question, and agreement with human respondents. As AAO cases lacked formal difficulty labels, we inferred based on the accuracy percentage of AAO respondents: least (≥80%), middle (79%-51%), and most difficult (≤50%). Questions referencing images were tagged as “image” cases. For these questions, GPT model inputs were aided with descriptions based on the image interpretations published in the AAO answer during an “image-aided” cycle. The “combination” runs combined raw text inputs for non-“image” queries with the “image-aided” input. Further analysis was conducted on the complexity of GPT model responses by examining the verbosity of the models’ responses based on output character length. We sought to determine if there was any correlation between the output length and the inherent difficulty of the question. Moreover, we conducted a segment-based analysis, categorizing questions into those pertaining to the anterior segment of the eye and those related to the posterior segment. Additionally, we differentiated between purely textual questions and those that referenced or were based upon images. Lastly, to gauge the alignment of the GPT models with human responses, we quantified the proportion of AAO respondents’ selections that matched the GPT models’ choices.

χ2 test, Fisher’s exact test, Student’s t-test, and one-way analysis of variance (ANOVA) were conducted where appropriate [11]. The homogeneity of variance was assessed using Levene’s statistic. Where absent, Welch’s ANOVA was completed. Correlation was calculated using Spearman’s coefficient. All statistical analyses were conducted using Statistical Package for the Social Sciences (SPSS) version 29 (IBM SPSS Statistics, Armonk, NY, USA), with p < 0.05 considered significant. Patients and the public were not involved in the design, conduct, reporting, or dissemination plans of the research.

## Results

GPT-4.0 performed significantly better than GPT-3.5 (75% versus 46%, p<0.01) (Table 1). With respect to subspecialty, GPT-3.5 was weakest for glaucoma, refractive, and uveitis, scoring only 25%. It performed most strongly on cornea/anterior segment (71%). With GPT-4.0, all categories had either equal or improved performance, with the most notable improvement observed for neuro-ophthalmology cases (100% versus 38%, p=0.03). However, uveitis and refractive questions remained the most challenging for GPT-4.0, with no discernible improvement from GPT-3.5. GPT-4.0 excelled in neuro-ophthalmology (100%), cornea/anterior segment (76%), and pediatric questions (82%).

**Table 1:** Comparison of GPT model accuracy on clinical case questions stratified by ophthalmic subspecialty Direct: AAO questions directly inputted, Images: only image supplemental questions, Image-aid: image supplemental questions with interpretation, Combination: direct AAO questions and image supplemental questions with interpretation Abbreviations: AAO: American Academy of Ophthalmology, GPT: Generative Pre-trained Transformer *p-values are statistically significant at p<0.05.

Category	GPT 3.5 Direct (number (%))	GPT 4.0 Direct (number (%))	p-value	GPT-3.5 Images (number (%))	GPT-4.0 Images (number (%))	p-value	GPT-3.5 Images-aided (number (%))	GPT-4.0 Images-aided (number (%))	p-value	GPT-3.5 Combination (number (%))	GPT-4.0 Combination (number (%))	p-value
Cornea/anterior segment	5/7 (71)	6/7 (86)	-	3/4 (75)	3/4 (75)	-	3/4 (75)	4/4 (100)	-	5/7 (71)	7/7 (100)	0.46
Glaucoma	1/4 (25)	3/4 (75)	0.49	0/1 (0)	0/1 (0)	-	0/1 (0)	1/1 (100)	-	1/4 (25)	4/4 (100)	0.14
Neuro-ophthalmology	3/8 (38)	8/8 (100)	0.03*	2/3 (67)	3/3 (100)	-	2/3 (67)	3/3 (100)	-	3/8 (38)	8/8 (100)	0.03*
Ocular pathology/oncology	1/3 (33)	2/3 (67)	-	1/3 (33)	2/3 (67)	-	1/3 (33)	2.3 (67)	-	1/3 (33)	2/3 (67)	-
Oculoplastic/obit	3/4 (75)	3/4 (75)	-	2/3 (67)	3/3 (100)	-	3/3 (100)	3/3 (100)	-	4/4 (100)	3/4 (75)	-
Pediatric	6/11 (55)	9/11 (82)	0.36	3/6 (50)	5/6 (83)	0.24	4/6 (67)	6/6 (100)	0.46	7/11 (64)	10/11 (91)	0.31
Refractive	1/4 (25)	1/4 (25)	-	0/1 (0)	0/1 (0)	-	0/1 (0)	1/1 (100)	-	1/4 (25)	2/4 (50)	-
Retina	3/7 (43)	5/7 (71)	0.59	3/7 (43)	5/7 (71)	0.59	5/7 (71)	5/7 (71)	-	5/7 (71)	5/7 (71)	-
Uveitis	1/4 (25)	1/4 (25)	-	0/2 (0)	1/2 (50)	-	1/2 (50)	1/2 (50)	-	1/4 (25)	2/4 (50)	-
Overall	24/52 (46)	39/52 (75)	<0.01*	14/30 (47)	22/30 (73)	0.07	19/30 (63)	26/30 (87)	0.07	28/52 (54)	43/52 (83)	<0.01*

Among the 30 (58%) image-related questions, GPT-4.0 demonstrated greater accuracy than GPT-3.5 (73% versus 46%, p=0.07). There was no notable difference between the two models in categorical performance. The performance gap between the models remained consistent when they were provided with image interpretations. GPT-3.5 improved its accuracy by 14%, while GPT-4.0 improved by 16%. When presented with a combination of text-based questions and image questions with interpretations, GPT-4.0 still outperformed GPT-3.5 by a large margin (83% versus 54%, p<0.01) with a significant improvement for neuro-ophthalmology cases (p=0.03). Examples of the GPT models’ inputs and outputs for the text-only and image questions can be found in Table 2.

**Table 2:** Sample AAO question inputs and GPT model’s output Abbreviations: AAO: American Academy of Ophthalmology, GPT: Generative Pre-trained Transformer

Input Type	AAO Difficulty	Input	GPT-3.5 Output	GPT-3.5 Outcome	AAO Agreement	GPT-4.0 Output	GPT-4.0 Outcome	AAO Agreement
Text	86% (least)	A 3-month-old exhibits bilateral eye irritation, with complaints of chronic, mostly clear tearing and occasional lash mattering. The exam is otherwise unremarkable, including the presence of normal intraocular pressure. What is the most appropriate initial management? Topical antibiotic-steroid eye drops, dacryocystorhinostomy, observation and digital massage, nasolacrimal duct probing	The most appropriate initial management for a 3-month-old with bilateral eye irritation and complaints of chronic, mostly clear tearing and occasional lash mattering would be observation and digital massage. In infants, excessive tearing and eye irritation can often be caused by nasolacrimal duct obstruction, which is a common condition that occurs when the nasolacrimal duct, a tube that carries tears from the eye to the nose, becomes blocked. Nasolacrimal duct obstruction can be caused by a variety of factors, including inflammation, infection, or structural abnormalities. Observation and digital massage can be effective in relieving the symptoms of nasolacrimal duct obstruction in infants. Digital massage involves gently massaging the area around the inner corner of the eye, where the nasolacrimal duct is located, in order to help clear the blockage. If the symptoms resolve with digital massage, further treatment may not be necessary. If the symptoms do not improve with digital massage, or if the infant has other signs or symptoms that suggest a more serious underlying condition, further testing or treatment may be necessary. Topical antibiotic-steroid eye drops would not be the most appropriate initial management in this case, as there is no evidence of infection or inflammation on exam. Dacryocystorhinostomy and nasolacrimal duct probing are surgical procedures that are typically reserved for cases of nasolacrimal duct obstruction that do not respond to non-surgical treatment, such as digital massage.	Correct	86%	In a 3-month-old with bilateral eye irritation, chronic clear tearing, and occasional lash mattering, the most appropriate initial management would be observation and digital massage. These symptoms are suggestive of nasolacrimal duct obstruction, which is relatively common in infants and often resolves spontaneously within the first year of life. Parents can be instructed to perform digital massage, also known as the “​​​Crigler massage,”​​​​​​​ which involves applying gentle pressure over the nasolacrimal sac area to facilitate the opening of the obstruction. If the symptoms persist or worsen, further intervention such as nasolacrimal duct probing may be considered.	Correct	86%
Text	29% (most)	Several years ago, a patient had a small-aperture corneal inlay implanted to treat presbyopia, as shown. The patient has since developed diabetic retinopathy requiring intervention. What procedure is compromised by the presence of the inlay? Applanation tonometry, focal laser to the macula, injection of vascular endothelial growth factor (VEGF) medications, fundus photography	The presence of a small-aperture corneal inlay may compromise the accuracy of applanation tonometry, a procedure used to measure intraocular pressure (IOP) in the eye. Applanation tonometry involves the application of a small, flat pressure-sensing device to the surface of the cornea. The inlay, which is a thin, opaque disk that is implanted in the cornea to change its refractive power and treat presbyopia, can affect the way the cornea flattens under pressure and, therefore, may interfere with the accuracy of IOP measurements. The presence of the inlay should not compromise focal laser to the macula, injection of vascular endothelial growth factor (VEGF) medications, or fundus photography. These procedures do not involve contact with the inlay or the front surface of the cornea. It is important to carefully consider the potential impact of any ocular implants or devices on diagnostic or therapeutic procedures, and to take appropriate precautions as needed. In the case of a small-aperture corneal inlay, it may be necessary to use an alternative method of IOP measurement or to adjust the measurement technique to account for the presence of the inlay.	Incorrect	38%	The presence of a small-aperture corneal inlay can compromise the accuracy of applanation tonometry. Applanation tonometry is used to measure intraocular pressure (IOP), and the presence of the corneal inlay can alter the corneal biomechanics, leading to inaccurate IOP measurements. Focal laser to the macula, injection of vascular endothelial growth factor (VEGF) medications, and fundus photography are typically not compromised by the presence of a corneal inlay, as these procedures target the posterior segment of the eye and are not directly affected by the presence of the inlay in the cornea.	Incorrect	38%
Image	46% (most)	A 60-year-old woman complains of difficulty reading. She is under treatment for fibromyalgia, arthritis, interstitial cystitis, and hypertension. She has no family with retinal disease. Her visual acuity is 20/20 in each eye with macular appearance and fundus autofluorescence as shown. What is the most likely diagnosis? Retinal toxicity associated with pentosan polysulfate sodium, age-related macular degeneration, pattern dystrophy, retinal toxicity associated with hydroxychloroquine	Based on the information provided, the most likely diagnosis is age-related macular degeneration (AMD). AMD is a common condition that affects the central portion of the retina, called the macula, which is responsible for detailed vision such as reading and driving. It typically occurs in people over the age of 50 and is more common in those with a family history of the condition. The patient’s age and the presence of macular degeneration are consistent with the diagnosis of AMD. The other conditions listed (retinal toxicity associated with pentosan polysulfate sodium, pattern dystrophy, and retinal toxicity associated with hydroxychloroquine) are not as likely based on the information provided. It is important to perform additional testing (such as imaging studies and laboratory tests) to confirm the diagnosis and determine the appropriate course of treatment.	Incorrect	12%	The image is not provided, but based on the description provided, the patient is experiencing difficulty reading and is under treatment for several conditions, including fibromyalgia, arthritis, interstitial cystitis, and hypertension. Considering her medical history and the absence of any family history of retinal disease, the most likely diagnosis is: 1. Retinal toxicity associated with pentosan polysulfate sodium: This medication is used to treat interstitial cystitis and has been associated with a risk of retinal toxicity, which can present with difficulty reading and changes in the macular appearance. Other differential diagnoses mentioned include: 2. Age-related macular degeneration (AMD): Although the patient is 60 years old, the absence of family history and her relatively good visual acuity of 20/20 in each eye make AMD less likely in this case. 3. Pattern dystrophy: This is a group of inherited retinal diseases, but the patient has no family history of retinal disease, which makes this diagnosis less likely. 4. Retinal toxicity associated with hydroxychloroquine: This medication is used for treating various autoimmune conditions, such as rheumatoid arthritis and lupus. However, the patient’s medical history doesn’t mention the use of hydroxychloroquine. In summary, given the patient’s medical history, the most likely diagnosis is retinal toxicity associated with pentosan polysulfate sodium.	Correct	46%
Image-aided	46% (most)	A 60-year-old woman complains of difficulty reading. She is under treatment for fibromyalgia, arthritis, interstitial cystitis, and hypertension. She has no family with retinal disease. Her visual acuity is 20/20 in each eye with macular appearance and fundus autofluorescence as shown. Macular findings include pigmented parafoveal deposits. What is the most likely diagnosis? Retinal toxicity associated with pentosan polysulfate sodium, age-related macular degeneration, pattern dystrophy, retinal toxicity associated with hydroxychloroquine	Based on the information provided, it seems that the patient is experiencing difficulty reading and has macular findings that include pigmented parafoveal deposits. These findings are consistent with age-related macular degeneration (AMD), which is a common cause of visual impairment in older adults. AMD is characterized by the accumulation of deposits in the macula, which can lead to vision loss. Retinal toxicity associated with pentosan polysulfate sodium or hydroxychloroquine would not be the most likely diagnosis in this case, as there is no mention of the patient taking these medications. Pattern dystrophy is a rare genetic disorder that can cause vision loss, but it typically presents with characteristic patterns of retinal pigment epithelial (RPE) abnormalities, which are not described in this case. In conclusion, the most likely diagnosis in this case is age-related macular degeneration. Further testing, such as imaging studies and visual field testing, may be helpful in confirming the diagnosis and determining the appropriate management plan.	Incorrect	12%	The patient’s macular findings and her history of interstitial cystitis treatment suggest the diagnosis of retinal toxicity associated with pentosan polysulfate sodium. Pentosan polysulfate sodium is a medication commonly used for the treatment of interstitial cystitis, a chronic condition causing bladder pain and pressure. Studies have suggested a link between the long-term use of pentosan polysulfate sodium and a unique form of retinal toxicity, which can lead to vision changes. Age-related macular degeneration typically presents with drusen and changes in the retinal pigment epithelium, pattern dystrophy usually has a family history and specific patterns on autofluorescence, and retinal toxicity associated with hydroxychloroquine typically presents with parafoveal loss of the ellipsoid zone and "flying saucer" sign on OCT, which does not seem to be the case here.	Correct	46%
Image	77% (middle)	A 16-year-old girl presents with painless decreased vision in her right eye. She denies any history of head or eye trauma. Her slit-lamp exam is shown. What consultation should you order next? Rheumatology, neurology, cardiology, nephrology	Based on the information provided, the most appropriate consultation to order next would be with a rheumatology specialist. The slit-lamp examination shows the presence of a white, fluffy, and diffuse infiltrate in the anterior chamber of the eye, which is consistent with the diagnosis of uveitis. Uveitis is an inflammation of the uveal tract, which includes the iris, ciliary body, and choroid. It can be caused by a variety of factors, including autoimmune disorders, infections, and other underlying systemic conditions. Rheumatology is a subspecialty of internal medicine that focuses on the diagnosis and treatment of autoimmune and inflammatory conditions. A rheumatology consultation would be appropriate to help determine the cause of the patient’s uveitis and to guide management. While neurology, cardiology, and nephrology are all important specialties, they are not specifically indicated in this case based on the information provided. It is important to accurately diagnose and manage uveitis to prevent potential complications, such as vision loss or glaucoma.	Incorrect	13%	Given the very limited information, it’s challenging to make an accurate choice. However, considering the patient’s age and the common causes of painless vision loss in this age group, I would lean towards a neurology consultation. This is because neurological conditions like optic neuritis or a mass lesion (such as a brain tumor) pressing on the optic nerve can cause painless vision loss and may be more common in this age group. But remember, without more specific information, this is a guess and in a real clinical scenario, more information would be needed to make an appropriate referral.	Incorrect	2%
Image-aided	77% (middle)	A 16-year-old girl presents to the ophthalmologist with painless decreased vision in her right eye. She denies any history of head or eye trauma. Her slit-lamp exam is shown, with lens subluxation. Which one of the following consultations should you order next? Rheumatology, neurology, cardiology, nephrology	Based on the information provided, the most appropriate consultation to order would be with a rheumatologist. Lens subluxation, or displacement of the lens within the eye, can sometimes be a symptom of an underlying systemic condition such as rheumatoid arthritis or Marfan syndrome. A rheumatologist is a specialist who can evaluate the patient for these and other rheumatologic conditions that may be causing the lens subluxation. It is important to identify and address any underlying systemic conditions in order to properly manage the lens subluxation and ensure the best possible outcome for the patient’s vision.	Incorrect	13%	The patient has lens subluxation, which is a hallmark feature of Marfan syndrome - a connective tissue disorder that affects many organ systems, including the skeletal, cardiovascular, and ocular systems. Marfan syndrome is an autosomal dominant disorder caused by mutations in the fibrillin-1 gene. Cardiovascular involvement, particularly aortic root dilatation leading to aortic dissection, is the most serious manifestation of Marfan syndrome and the main cause of premature death. Thus, the most urgent next step in management is a cardiology referral for echocardiographic evaluation of the aorta. It’s important to initiate appropriate treatment and monitoring to prevent cardiovascular complications.	Correct	77%

When the questions were categorized by difficulty according to the percentage of AAO respondents who answered each correctly, GPT-3.5 did not show any difference in accuracy across difficulty levels (p=0.30), while GPT-4.0 performed better on easier questions relative to harder ones (94% (most difficult) versus 76% (middle) versus 53% (least), p=0.03) (Table 3). There was no significant difference between the two models in terms of accuracy or response length for anterior versus posterior segment questions or image versus text-only questions. Overall, GPT-4.0 generated shorter answers than GPT-3.5 (651.7±342.9 versus 1,112.9±328.8 characters, p<0.01). The official AAO explanations were longer than GPT-4.0’s answers by 310.2±147.5 characters on average (p<0.01).

**Table 3:** GPT model analysis based on response complexity (character length) and AAO respondent agreement Complexity analysis was performed using character counts excluding spaces. AAO agreement was reported as % of AAO respondents who chose the same answer as the GPT model. Abbreviations: AAO: American Academy of Ophthalmology, SD: standard deviation, GPT: Generative Pre-trained Transformer †Comparison of GPT-3.5 and GPT-4.0 ‡Comparison of GPT-4.0 to AAO responses *p-values are statistically significant at p<0.05.

	Least difficult (≥80%) (n=16)	Middle (n=21)	Most difficult (≤50%) (n=15)	p-value	Anterior segment (n=30)	Posterior segment (n=22)	p-value	Text-only (n=23)	Image (n=29)	p-value	Total (n=52)	p-value
Question length (mean±SD)	362.3±131.0	407±157.6	408.5±155.3	0.60	380.0±147.2	412.7±150.0	0.44	429.0±159.5	365.9±134.2	0.13	393.9±147.8	-
GPT-3.5 accuracy (number (%))	10 (63)	8 (38)	6 (40)	0.30	16 (53)	8 (36)	0.23	10 (44)	14 (48)	0.73	24 (46)	<0.01^†^
GPT-4.0 accuracy (number (%))	15 (94)	16 (76)	8 (53)	0.03*	22 (73)	17 (77)	0.75	22 (76)	17 (74)	0.87	39 (75)	
GPT-3.5 response length (mean±SD)	1,104.6±426.0	1,165±263.1	1,081.6±296.7	0.75	1,102.0±331.1	1,127.9±332.9	0.78	1,070.4±269.8	1,146±370.3	0.41	1,112.9±328.8	<0.01 ^†^
GPT-4.0 response length (mean±SD)	555.4±133.1	675.0±410.6	721.8±389.8	0.38	624.7±304.5	688.5±393.8	0.53	568.0±256.9	718.0±389.9	0.10	651.7±342.9	
AAO response length (mean±SD)	847.4±439.4	1,024.5±524.5	996.3±504.0	0.53	888.4±534.6	1062±413.5	0.21	1049.6±548.8	892.3±435.9	0.26	961.9±490.4	<0.01 ^‡^
GPT-3.5 AAO agreement (mean±SD) (%)	57±41	36±27	32±18	0.11	47±34	34±27	0.16	41±32	43±31	0.41	41±31	<0.01^†^
GPT-4.0 AAO agreement (mean±SD) (%)	83±19	52±3	37±17	<0.01*	62±31	51±23	0.19	60±27	55±29	0.50	57±28	

GPT-4.0 performed better than GPT-3.5 in generating answers that matched with AAO respondents (57%±28% versus 41%±31%, p<0.01). GPT-3.5 had similar agreement rates regardless of question difficulty, while GPT-4.0 had higher agreement rates for easier questions and lower agreement rates for harder questions (83% (most difficult) versus 52% (middle) versus 37% (least), p<0.01). Additionally, there was a strong correlation between GPT-4.0’s accuracy and the proportion of AAO respondents who selected GPT-4.0’s answer (ρ=0.713, p<0.01). The agreement rates of both models did not vary by segment or question type.

## Discussion

Our study highlights significant advancements in the application of AI for ophthalmology, particularly in the performance metrics between GPT-4.0 and GPT-3.5. Specifically, GPT-4.0 significantly outperformed GPT-3.5 in overall accuracy and in the neuro-ophthalmology subspecialty, as detailed in Table 1. Equally important, GPT-4.0 showed strength in handling image-related questions, maintaining its advantage even when supplied with image interpretations, as shown in Table 3.

The effectiveness of NLP models in addressing AAO clinical scenarios is further evidenced by their improving track record on ophthalmic board practice questions [7,9,12]. Mihalache et al.’s study highlighted a significant improvement in ChatGPT’s performance on the OphthoQuestions question bank for OKAP examinations, increasing from 46% to 58% over a one-month period [7]. Remarkably, within just a few months after the release of GPT-4.0, it achieved an average accuracy of 71.6% in ophthalmology, nearly matching the human respondents’ accuracy of 72.2% in a dataset of 250 questions [9]. The advancements in NLP model performance in specialized knowledge are further corroborated by a neurosurgery study that assessed GPT-4.0’s performance on oral board examinations; here, it achieved an impressive accuracy rate of 82.6%, far exceeding GPT-3.5’s 62.4% [13]. This upward trajectory underscores the dynamic evolution of NLP models, which are becoming robust and reliable in a relatively short period. Our study, covering ophthalmology clinical case questions, reaffirms this rapid advancement, emphasizing the speed at which conversational AI systems are mastering complex, specialized tasks.

OpenAI’s release of GPT-4.0 brought several improvements. When applied to specialized domains such as ophthalmology, these advancements lead to increased accuracy. One of the most notable changes is the sheer scale. The original GPT model had 117 million parameters, while GPT-3.0 had 175 billion parameters, and GPT-4.0 boasts a staggering 170 trillion [14]. The massive increase in model size allowed for the later models to store more information and recognize more nuanced patterns. Using more extensive and diverse datasets, including books, articles, and websites, the GPT models capture a broader spectrum of ophthalmic knowledge. Aside from the scale of training, newer models had more advanced training methodologies. With GPT-3.0, there was the introduction of few-shot learning. Unlike traditional machine learning models that require extensive labeled data for specific tasks, the most recent GPT systems can employ meta-learning to complete new tasks based on the pattern it identifies from a limited number of examples [15]. A recent study has shown that GPT-4.0 increasingly excels at zero-shot learning and can perform tasks with no prior training [16]. This process leverages semantic relationships between categories. For instance, if the model knows that a zebra is like a horse and has stripes, it will still be able to recognize one, even if it has never seen the animal. Although OpenAI explicitly mentioned in their GPT-4.0 technical report that they would not disclose the exact internal architecture, potentially due to the growing competition in the AI field, they did provide some insights into the updates from their previous model. OpenAI indicates that GPT-4.0 introduced a rule-based reward model (RBRM), complementing the reinforcement learning with human feedback (RLHF) of GPT-3.5 [8]. The RBRM approach enhances the fine-tuning process by ranking various system responses and providing a reward signal to the top output, based not only on user feedback but also on pre-set parameters. This ensures the language model’s compliance with generating correct content and reducing “hallucinations,” unwarranted confident answers [17,18]. Furthermore, the context window length of GPT-4.0 is 4-6 times greater than GPT-3.5 [8]. The context width refers to the number of previous tokens or words the model uses to formulate its response, allowing for greater relevance, coherence, and quality in its outputs. This enhanced contextual understanding boosts the neural pathways’ capacity to detect intricate patterns, which can help interlink symptoms or systemic conditions.

Regarding the conciseness of GPT-4.0’s answers, this attribute may have significant practical implications. With the widespread use of practice questions among medical and proven associations between the number of completed practice questions and board examination performance [19], the ability to provide explanations to students gives NLP an applicable role in medical education. With comparatively shorter responses to both ChatGPT 3.5 and the AAO-provided explanation, ChatGPT 4.0’s changes could translate into succinct feedback for students looking for abbreviated explanations. Moreover, more concise responses could indicate better computational efficiency in data processing, saving both time and resources [20]. Furthermore, the increased alignment of GPT-4.0’s answers with those of human respondents from the AAO is particularly promising. This greater congruence not only suggests that GPT-4.0 is becoming more attuned to medical consensus and clinical reasoning pathways [21]. The enhanced alignment of GPT-4.0’s responses with those of AAO human respondents suggests an increasing congruence with medical consensus and established clinical reasoning, raising the possibility that future versions of the GPT series could be even more closely aligned with clinical best practices.

While the rapid evolution of the GPT models offers substantial benefits for medical training, there are risks of misinformation. As highlighted in Table 2, GPT models are prone to “hallucinations,” which could lead to misdiagnosis if not caught by a knowledgeable expert [9,17]. These instances question the reliability of the model, particularly where precise clinical decisions are essential. Additionally, the differential performance of GPT-4.0 across varying levels of question difficulty illuminates nuanced challenges that constrain their utility in comprehending and responding to ophthalmology cases. While GPT-4.0 performs admirably on straightforward, single-step questions, its decreased efficacy with image or increasingly complex queries potentially points to limitations in multistep reasoning and inference, as noted by Cai et al. [9]. The viability of GPT models in an inherently visual field such as ophthalmology is hampered by their inability to natively process image data [12]. This is particularly concerning for real-world clinical applications, where medical decision-making often involves a labyrinth of interrelated variables, from patient histories and diagnostic imaging to multifaceted treatment algorithms. Furthermore, the model’s struggle with image questions that require inference highlights its shortcomings for nuanced clinical settings, where physicians routinely make probabilistic decisions based on ambiguous visual data [20]. While GPT-4.0 may serve as a resource for answering basic queries, reliance on it for more complex scenarios or visual-based diagnostics would be premature. Although our analysis did not evaluate biases in these models, there are known shortcomings in GPT models. Inherent biases in source materials or the data selection process can bias AI models. Additionally, the algorithms and data labeling for AI learning may introduce or emphasize certain features or data points [22]. This becomes especially problematic in healthcare, where biased models can lead to unequal treatment for specific patient groups. Training these models on data predominantly sourced from particular populations might associate certain diseases with specific demographic factors, furthering potentially detrimental stereotypes. Despite the advancements of AI, it is critical to consider these limitations and use these models as complementary resources rather than absolute substitutes for human expertise.

While our methodology was designed to broadly assess GPT performance, the investigation possessed certain limitations. Most notably, the use of multiple-choice questions does not fully capture the intricate dynamics of clinical decision-making. Although these types of questions can test factual knowledge effectively, these questions oversimplify scenarios and only present the relevant parts of the presentation. In practice, physicians do not simply select from predefined options. They must consider a broad spectrum of symptoms, patient history, and individual factors that may affect potential outcomes. This depth and complexity of a patient necessitate an evaluation of the patient in its entirety. Clinicians then parse out key clinical findings that would lead to the appropriate diagnosis and treatment plan. This level of nuanced understanding and experience cannot be fully tested by this multiple-choice format. Moreover, the restricted pool of post-training data AAO cases made it difficult to conduct a granular analysis of the GPT models’ performance across different types of questions. This limitation is particularly relevant when considering the possibility that these models might excel or falter based on specific question categories.

## Conclusions

GPT-4.0 outperformed its predecessor GPT-3.5 in answering the AAO “Diagnose This” set of multiple-choice ophthalmic clinical cases, especially in neuro-ophthalmology, with improved accuracy even in image-related questions. Within the span of a couple of months, these vastly improved GPT-4.0 results not only signify the rapid pace of technological advancement in machine learning but also underscore the transformative potential AI holds for affecting ophthalmic student education and medical practice. AI models could serve as a supplementary educational tool for ophthalmology students, providing instantaneous, data-driven feedback that could enrich traditional learning environments. However, it is not without limitations, including the risk of generating fictional information and weakness in more complex inference-based cases. Future studies should not only focus on accuracy but also assess the level of concordance between AI response rationale and expert consensus. This could help in refining the algorithms and ensuring they align more closely with established medical knowledge and clinical best practices.

As we contemplate the increasing role of AI in medicine, it is crucial to remember the ethical and professional obligations that physicians carry. Technology should be seen as a complement to, not a replacement for, the expert. Doctors are ultimately responsible for patient outcomes, and thus, they need to be cautious and selective in the kinds of technology they incorporate into their practice and training regime.
